# “Oxygen Sensing” by Na,K-ATPase: These Miraculous Thiols

**DOI:** 10.3389/fphys.2016.00314

**Published:** 2016-08-02

**Authors:** Anna Bogdanova, Irina Y. Petrushanko, Pablo Hernansanz-Agustín, Antonio Martínez-Ruiz

**Affiliations:** ^1^Institute of Veterinary Physiology, Vetsuisse Faculty and the Zurich Center for Integrative Human Physiology (ZIHP), University of ZurichZurich, Switzerland; ^2^Engelhardt Institute of Molecular Biology, Russian Academy of SciencesMoscow, Russia; ^3^Servicio de Inmunología, Instituto de Investigación Sanitaria Princesa (IIS-IP), Hospital Universitario de La PrincesaMadrid, Spain; ^4^Departamento de Bioquímica, Universidad Autónoma de MadridMadrid, Spain

**Keywords:** Sodium-Potassium-Exchanging ATPase, redox regulation, thiols, hypoxia, S-glutathionylation, S-nitrosylation

## Abstract

Control over the Na,K-ATPase function plays a central role in adaptation of the organisms to hypoxic and anoxic conditions. As the enzyme itself does not possess O_2_ binding sites its “oxygen-sensitivity” is mediated by a variety of redox-sensitive modifications including S-glutathionylation, S-nitrosylation, and redox-sensitive phosphorylation. This is an overview of the current knowledge on the plethora of molecular mechanisms tuning the activity of the ATP-consuming Na,K-ATPase to the cellular metabolic activity. Recent findings suggest that oxygen-derived free radicals and H_2_O_2_, NO, and oxidized glutathione are the signaling messengers that make the Na,K-ATPase “oxygen-sensitive.” This very ancient signaling pathway targeting thiols of all three subunits of the Na,K-ATPase as well as redox-sensitive kinases sustains the enzyme activity at the “optimal” level avoiding terminal ATP depletion and maintaining the transmembrane ion gradients in cells of anoxia-tolerant species. We acknowledge the complexity of the underlying processes as we characterize the sources of reactive oxygen and nitrogen species production in hypoxic cells, and identify their targets, the reactive thiol groups which, upon modification, impact the enzyme activity. Structured accordingly, this review presents a summary on (i) the sources of free radical production in hypoxic cells, (ii) localization of regulatory thiols within the Na,K-ATPase and the role reversible thiol modifications play in responses of the enzyme to a variety of stimuli (hypoxia, receptors' activation) (iii) redox-sensitive regulatory phosphorylation, and (iv) the role of fine modulation of the Na,K-ATPase function in survival success under hypoxic conditions. The co-authors attempted to cover all the contradictions and standing hypotheses in the field and propose the possible future developments in this dynamic area of research, the importance of which is hard to overestimate. Better understanding of the processes underlying successful adaptation strategies will make it possible to harness them and use for treatment of patients with stroke and myocardial infarction, sleep apnoea and high altitude pulmonary oedema, and those undergoing surgical interventions associated with the interruption of blood perfusion.

## Introduction. oxygen, and oxygen sensing from evolutionary and modern perspectives

### Oxygen, redox state, ions, energy, and Na,K-ATPase

Sustaining of life is a process requiring high energy costs. Energy production in living systems utilizes transmembrane electrochemical gradients, those for ions and redox equivalents. No gradients can be sustained without a membrane, so membranes are the key elements of any living cell since the first proto-cell, the last universal common ancestor (LUCA), that existed more than 3 billion years ago on our planet (Sousa et al., 2013). As life evolved, more specialized compartments were formed within cells, each surrounded by its own membrane. Modern plasma membrane uses the energy of ATP produced by aerobic or anaerobic pathways to generate transmembrane Na^+^/K^+^ and Ca^2+^ gradients. One member of the P-type ATPases family, Na,K-ATPase, transforms the energy of phosphate bonds within the ATP to the energy of transmembrane Na/K gradient that is used to support the excitability of neurons and myocytes, control of intracellular Ca^2+^ levels and pH, intake of amino acids and fuel, and for sensing and signaling (Blanco and Mercer, 1998; Therien and Blostein, 2000; Blanco, 2005; Geering, 2006; Toyoshima et al., 2011; Reinhard et al., 2013; Shattock et al., 2015). It uses 1 ATP molecule to exchange 3 intracellular Na^+^ ions for 2 extracellular K^+^ ions per cycle. The number of cycles varies between 1500 and 10,000 per min (Liang et al., 2007) and the corresponding energy expenditure ranges between 20% of total energy consumption in non-excitable cells to 75% in excitable tissues under hypoxic conditions (Buck and Hochachka, 1993; Erecinska and Silver, 2001). Being a major sink for ATP under hypoxic conditions, Na,K-ATPase is capable of “sensing” the changes in O_2_ availability and adjusting its activity to the rates of ATP production (Bogdanova et al., 2006). This review summarizes the progress in our understanding of the mechanisms utilized by the Na,K-ATPase for “O_2_ sensing.” Recent developments in of the field of free radical biology and medicine have provided decisive clues for dissection of these mechanisms and the role that protein thiols play in it.

### Reactive oxygen (ROS) and nitrogen (RNS) species as signal messengers

Oxygen is a key component of the cell energy production machinery driving Cambrian explosion ~500 Mio years ago. It plays a role of the final acceptor of the mitochondrial electron transport chain (ETC), which is coupled to ATP production by the H^+^-dependent reversible mitochondrial ATPase in the mitochondrial oxidative phosphorylation (OXPHOS) system. Many other metabolic reactions in the cell also use oxygen as a necessary component. Thus, cells have developed systems that can sense fluctuations in oxygen concentration and perform different adaptations, and there are several links between hypoxia sensing and redox reactions that we briefly explore here.

**Figure 1:**
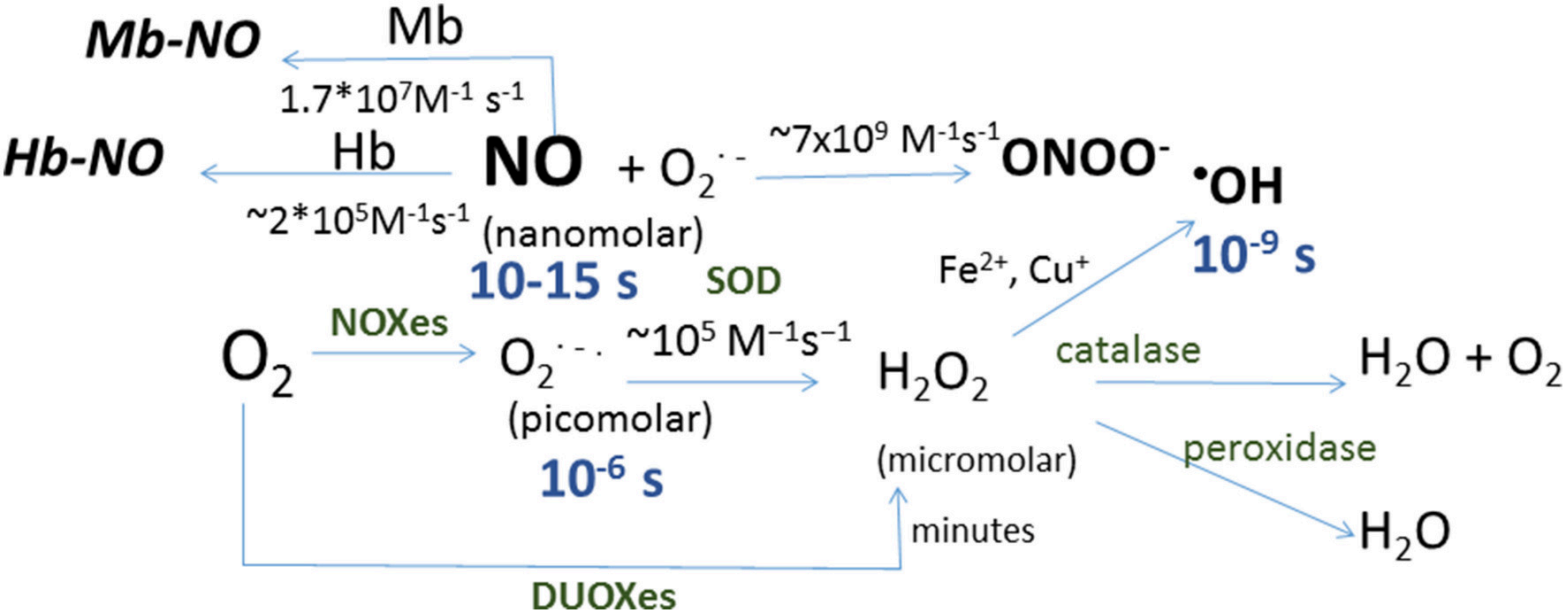
**Schematic representation of reactions in which reactive oxygen and nitrogen species are formed**. Shown in blue are the half-life for each species and in brackets the concentration range for each species in biological systems, and in green the enzymes catalyzing the corresponding reactions: dual oxidizes (DOUXes), superoxide dismutases (SOD). Under the arrows are the rate constants for the reactions shown. Myoglobin (Mb) and hemoglobin (Hb) are shown as sinks for NO.

Oxygen produces reactive oxygen species (ROS) by successive one-electron reductions (Figure 1). Superoxide anion (${\text{O}}_{2}^{•-}$) is the first ROS formed from O_2_; its half-life is as short as 2 × 10^5^ M^−1^s^−1^ (Kalyanaraman, 2013), what makes ${\text{O}}_{2}^{•-}$ particularly difficult to detect. It is not a powerful oxidant, and its effect on protein thiols is mainly limited to disruption of iron-sulfur clusters; in this regard, mitochondrial aconitase inactivation has been a classical hallmark of mitochondrial superoxide production (Fridovich, 1995; Hausladen and Fridovich, 1996). It dismutates spontaneously or by the action of cytosolic or mitochondrial superoxide dismutases (Cu,Zn-SOD or Mn-SOD respectively), producing a reduced form, hydrogen peroxide (H_2_O_2_), and an oxidized form, water (H_2_O). Hydrogen peroxide half-life is quite greater than that of its predecessor, 10^3^–10^4^ M^−1^s^−1^ (Kalyanaraman, 2013), it can cross biological membranes and oxidize thiols within Cys residues in proteins. The latter capacity has been a useful tool for nature to design molecular sensors of H_2_O_2_; likewise several laboratories have developed different fluorescent proteins capable of detecting H_2_O_2_ through reversible oxidation of critical, sensitive thiols in their structure (Hanson et al., 2004; Belousov et al., 2006; Ermakova et al., 2014). All together, these features make H_2_O_2_ the most easily detectable ROS and the best known. A one-electron reduction of hydrogen peroxide forms hydroxyl radical (^•^OH), a reaction that can be catalyzed by the oxidation of Fe^2+^ into Fe^3+^ in the so-called Fenton reaction (Kalyanaraman, 2013). ^•^OH is the most reactive and probably the most toxic free radical due to the initiation of radical chain reactions. It is worth mentioning the very fast reaction of superoxide with nitric oxide (NO^•^) to form peroxynitrite (ONOO^−^), with a rate comparable to that of diffusion, 4–6 × 10^9^ M^−1^s^−1^ (Kalyanaraman, 2013). Although peroxynitrite (one of the reactive nitrogen species, RNS) is well known as an inducer of tyrosine nitration, it is a peroxide and as such it is a very effective two-electron oxidant of thiols.

### ROS/RNS and hypoxia

Since ROS are chemical derivatives of O_2_, hypoxia would lower the production of ROS due to the law of mass action, i.e., as there is a lower oxygen concentration, one would expect to observe a decrease in ROS production. However, cell systems are often more complicated than the basic chemical systems in a test tube with multiple players and reactions involved in production and scavenging of ROS and RNS. Apart of thermodynamic and kinetic restrains, compartmentalization within the cell and species/cell type-specific settings affect the capacity of electron donors to react with the oxygen molecules and time course of these reactions. Indeed, there has been a long-standing controversy in the field, as there are a number of observations reporting lower ROS production in hypoxia (Hagen et al., 2003; Acker et al., 2006; Chua et al., 2010; Fernandez-Aguera et al., 2015) but also the opposite (Chandel et al., 1998, 2000; Guzy and Schumacker, 2006), what has been called the paradoxical ROS increase in hypoxia (Turrens, 2003; Guzy and Schumacker, 2006). This controversy emerged from the differences in experimental design including timing of hypoxic exposure prior to ROS detection, cell type, tissue or organism, as well as from the methodological diversity in ROS detection. For example, two groups detecting ${\text{O}}_{2}^{•-}$ in cells using superoxide-sensing probe (dihydroethidium) and microscopy reported the opposing findings (Quintero et al., 2006; Chua et al., 2010). The only decisive difference was the time of hypoxic exposure. Detection of ${\text{O}}_{2}^{•-}$ in cells following 3 h of hypoxia showed no changes in superoxide levels compared to the normoxic control (Chua et al., 2010), while facilitation of ${\text{O}}_{2}^{•-}$ production was observed during the first hour of hypoxia (Quintero et al., 2006). A recent report reconciled this apparent controversy, as it showed that production of mitochondrial superoxide is upregulated exclusively during the first minutes of hypoxia and its production decreases afterwards, in what has been called a superoxide burst in hypoxia (Hernansanz-Agustin et al., 2014).

Several sources have been proposed to be the origin of ROS in hypoxia. One of the first enzymes implicated in ROS generation in hypoxia was NADPH oxidase, which plays a role in hypoxic pulmonary vasoconstriction (HPV) (Marshall et al., 1996). Together with this, xanthine oxidase was also shown to produce ROS in hypoxia in neurons, contributing to cell death in ischemic conditions (Abramov et al., 2007).

Last but not least, mitochondria-derived ROS have been also shown to increase in hypoxia (Chandel et al., 1998, 2000; Hernansanz-Agustin et al., 2014), are necessary for ROS production by NADPH oxidase in hypoxic pulmonary vasoconstriction (Moreno et al., 2014) and are prior to the xanthine oxidase-derived ROS (Abramov et al., 2007). The source of electron leakage giving rise to an excessive production of ${\text{O}}_{2}^{•-}$ remains a matter of intensive investigation. Listed below are some considerations regarding the causes and possible mechanisms of hypoxia-inducible ${\text{O}}_{2}^{•-}$ burst in the mitochondria.

### ROS and RNS production in hypoxic mitochondria

Mitochondrial electron transport chain (mtETC) consists of four protein complexes within the mitochondrial inner membrane. Complexes I and II oxidize electron carriers NADH and FADH_2_, and transfer the electrons to complex III by means of ubiquinone. Complex III reduces cytochrome *c*, which transports the electrons to complex IV and reduces O_2_ to H_2_O. These activities of complexes I, III, and IV are coupled to the pumping of H^+^ across the mitochondrial inner membrane, thus creating an electrochemical gradient. The oxidative phosphorylation system (OXPHOS) includes a fifth complex, complex V or ATP synthase that transforms the energy of proton gradient to phosphorylation of ADP to ATP (Mitchell, 1961; Mitchell and Moyle, 1967).

It is suggested that ROS generation occurs mainly at the level of complex I, but also at complex III (Turrens, 2003; Murphy, 2009; Drose and Brandt, 2012). FMN group within complex I oxidizes NADH into NAD^+^ and transfers electrons to a series of Fe-S clusters which, in turn, reduce ubiquinone into ubiquinol (Berrisford and Sazanov, 2009; Hunte et al., 2010; Zickermann et al., 2015) in the so-called forward electron transport. On the other hand, at high mitochondrial transmembrane potential ΔΨmt or in the presence of the complex II substrate succinate, complex I can accept electrons from ubiquinol and reduce NAD^+^ into NADH at the level of flavin mononucleotide FMN, together with the intrusion of H^+^ in a process called reverse electron transport (RET) (Vinogradov and Grivennikova, 2005, 2016; Drose and Brandt, 2012). In both forward and reverse transport there is a leakage of electrons giving rise to ${\text{O}}_{2}^{•-}$ production. Inhibitors targeting the ubiquinone-binding site (e.g., rotenone; Pryde and Hirst, 2011) increase the leakage in the forward transport, but inhibit it in RET. It is also reported in the presence of high concentrations of succinate, condition associated with progression of ischemia-reperfusion injury (Drose and Brandt, 2012; Chouchani et al., 2014). RET also takes place at elevated ΔΨmt (Korshunov et al., 1997; Drose and Brandt, 2012). ${\text{O}}_{2}^{•-}$ produced by complex I will be released toward the mitochondrial matrix where it is dismutated by Mn-SOD (SOD2) to H_2_O_2_. The latter is further detoxified by GSH-Glutaredoxin2 system within the matrix (Drose et al., 2014). Complex III can also give rise to ${\text{O}}_{2}^{•-}$ production along with oxidation of ubiquinol to ubiquinone known as Q cycle (Boveris et al., 1976; Cadenas et al., 1977). This process includes formation of semiquinone as an intermediate step (Trumpower, 1990), and this step becomes rate-limiting at high ΔΨmt or in the presence of the complex III-Qi site inhibitor Antimycin A. In case of electron leakage from complex III ${\text{O}}_{2}^{•-}$ is accumulating within the intermembrane space. After dismutation by Cu,Zn-SOD (SOD1) the resulting H_2_O_2_ is extruded from the mitochondria into the cytosol through porin (Drose et al., 2014) and then detoxified by catalase and GSH/glutaredoxin 2 systems. Free radicals and H_2_O_2_ originating from these two complexes most likely serve as local signaling messengers.

Evidence obtained using transgenic animal models suggest different mitochondrial complexes of the mtETC are involved in generation of superoxide anion participating in redox signaling. Knocking down Rieske iron-sulfur protein (RISP) within the complex III indicates the key role of electron transport by this complex for HIF-1α stabilization under hypoxic conditions (Brunelle et al., 2005; Guzy et al., 2005; Mansfield et al., 2005). Silencing of the NDUFS2 protein expression within complex I compromised hypoxia-induced ROS production and arterial chemoreception *in vivo* (Fernandez-Aguera et al., 2015). However, none of these studies explained the paradox of ROS production in hypoxia or addressed the mechanism of their production. Our recent data point to the key roles of complex I deactivation and Na^+^/Ca^2+^ exchange through the mitochondrial Na^+^/Ca^2+^ exchanger in the hypoxic superoxide production (Hernansanz-Agustín et al., manuscript submitted).

CO, H_2_S, and NO production are most likely supported within the mitochondria by heme oxygenase 1 (Ryter et al., 2006), mitochondrial NO synthase (Ghafourifar and Sen, 2007), and 3-mercaptopyruvate sulfurtransferase (Li et al., 2011). All gasses were shown to protect tissues from irreversible suppression of respiratory capacity of mitochondria during ischemia-reperfusion injury (Elrod et al., 2007). In addition to blocking complex III H_2_S is also capable of direct binding to the heme group of complex IV (Cooper and Brown, 2008). Supplementation of H_2_S has been shown to inhibit HIF-1α stabilization in hypoxic, but not in anoxic conditions, which is probably related to its capacity of inhibiting mitochondrial respiration (Kai et al., 2012). Systemic administration of sulfide was shown to sustain hibernation state (Blackstone et al., 2005). Nitrite causes inhibition of complex I by S-nitrosylation (reviewed in Martinez-Ruiz et al., 2011) and decreases free radical generation in tissues exposed to ischemia-reperfusion (Shiva et al., 2007).

Genetic adaptation to hypoxia via stabilization of the alpha subunit of hypoxia-inducible factors (HIF1α and HIF2α), has been suggested to require mitochondrial ETC ${\text{O}}_{2}^{•-}$ production (Chandel et al., 1998, 2000). However, how ROS act over HIFα subunits or its degrading enzymes, the prolyl-hydroxylases (EGLNs), is still unknown (Kaelin and Ratcliffe, 2008). More recently, mitochondrial complex I and ROS production have been shown to play a key role in acute oxygen sensing by carotid body (Fernandez-Aguera et al., 2015). Such increase in ROS production depolarizes glomus cells through inhibition of K^+^ channels and increase of cytosolic Ca^2+^ by extracellular Ca^2+^ influx. Both stabilization of HIF-α subunits and inhibition of K^+^ channels could be influenced by ROS through oxidation of thiols. Indeed, redox balance is also modified in hypoxia since ROS reversibly oxidize thiols in the cytosolic compartment of cells which, in turn, could have a role in cell signaling and survival during hypoxia (Izquierdo-Alvarez et al., 2012). Superoxide anion generation by mitochondria upon decrease in oxygenation below “normoxic values” as well as decline in NO production by neuronal and inducible NO synthases that have low affinity for O_2_ (K_d_ 2–5 kPa; Dweik, 2005) result in an increase in oxidized glutathione in hypoxic heart tissues of animals that are hypoxia-sensitive, but not in hypoxia-resistant ones (Petrushanko et al., 2012; Yakushev et al., 2012). Oxidized glutathione joins the reactions of non-enzymatic dithiol exchange in which S-glutathionylated adducts of thiol residues are formed in multiple proteins including the Na,K-ATPase. For mitochondrial ROS/RNS to be regulators of the Na,K-ATPase function under hypoxic conditions, the ROS generators should be co-localized with the ATPase. The existence of membrane-bound pool of mitochondria has been confirmed (Westermann, 2015). However, the role of ROS and RNS produced by the mitochondria in control of the Na,K-ATPase activity, amplifying and complementing the signals generated as NADPH oxidases, xanthine oxidase and other free radical generators, awaits further investigation.

Therefore, mitochondria-derived ROS produced in the first minutes of hypoxia (Hernansanz-Agustin et al., 2014), may be a primary and necessary event in redox signaling in hypoxia, leading to the activation of multiple redox processes. All these events converge to targeted thiol oxidation and development of acute adaptive response (Izquierdo-Alvarez et al., 2012).

Both mitochondria and Na,K-ATPase are corner-stones in control of metabolic state of hypoxic tissue. The intimate relation between them remains to be unraveled as it does not seem to be limited to the production and consumption of ATP alone.

## Versatility of oxygen sensing. multiple signals-multiple targets—multiple responses—multiple outcomes

Changes in O_2_ availability occurring in the course if evolution trigger multiple responses in every cell of any living organism on this planet (Holland, 2006). These responses are species- and cell type-specific. Diversity in responses matches the oxygen levels in the tissue under normoxic conditions (Bogdanova et al., 2006) and is associated with adaptations some species developed to survive hypoxic periods (Hochachka et al., 1999). A single protein, such as the Na,K-ATPase, may demonstrate essential hypoxia-insensitivity (Yakushev et al., 2012), maintain maximal activity within a narrow window of O_2_ concentrations (Petrushanko et al., 2007), or show linear dose-dependence of O_2_ concentration in the environment (Bogdanova et al., 2005; Yakushev et al., 2012) depending on the species and cell type. These responses also vary depending on the duration and severity of hypoxia (Dada et al., 2003; Fuller et al., 2003; Bogdanova et al., 2006; Petrushanko et al., 2007, 2015; Yakushev et al., 2012).

Patterns of response correlate with the changes in ROS/RNS and NO production in the cells and the corresponding shifts in redox state. Apart of the regulatory thiols within the Na,K-ATPase changes in the enzyme activity are in part mediated by its phosphorylation by multiple redox-sensitive kinases and phosphatases (Devarie-Baez et al., 2016). These versatile signaling messengers as well as the variability in free radical scavenging systems make responses to hypoxia dependent on location of free radical generators and speciation of the messengers (free radical- and gaseous-based). The mechanisms of oxygen-inducible regulation are largely restricted to the oxidative modifications of thiols (formation of mixed di-thiols with glutathione, sulfide or other protein thiols, S-nitrosylation, or proline/threonine carbonylation (Yan et al., 2013). These changes occur in multiple proteins at the same time. Fine-tuning of protein complexes is accomplished translating into the protection against hypoxia or reperfusion injury at the tissue and organism levels (Yan, 2014).

## Oxygen and redox-sensitivity of the Na,K-ATPase

### Na,K-ATPase and its thiols

**Figure 2:**
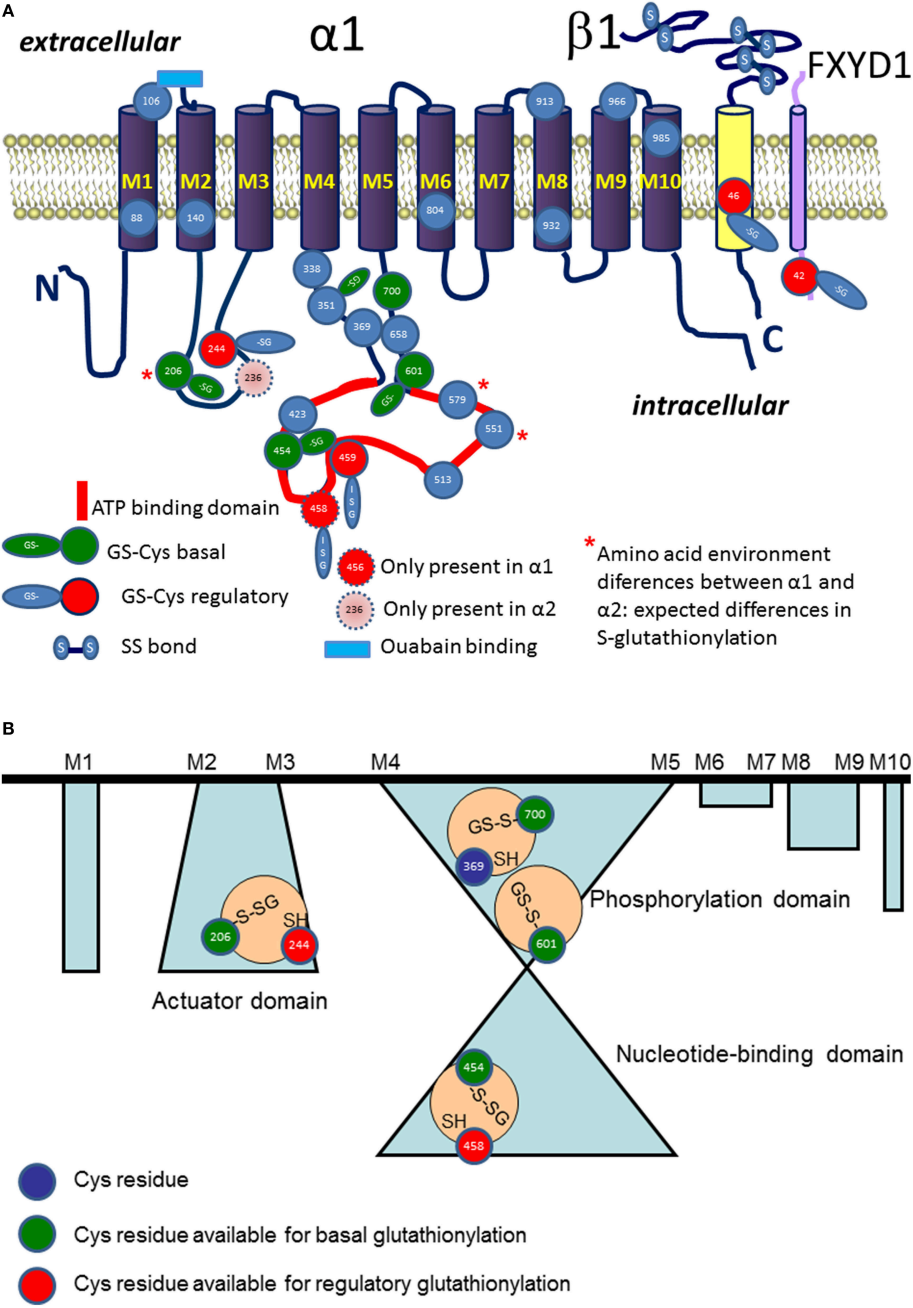
**Schematic representation of localization S-glutathionylated cysteine residues in α (in blue), β (in yellow), and phospholemman (FXYD, in cyan) subunits (A)**. Regulatory S-glutathionylation sites are shown in red. Basal S-glutathionylation is shown in green. ATP binding site is highlighted in red. Blue rectangle depicts ouabain binding site. Stars highlight the Cys residues with differences in pK between the α1 and α2 isoform (for details see Table 1). **(B)** Shows schematically the cavities with trapped S-glutathionylated Cys residues inaccessible for de-glutathionylation without detergents and representing “redox memory.”

Sodium potassium pump is formed by the 100–113 kDa catalytic α subunit, the regulatory obligatory 55 kDa β subunit and the tissue-specific regulatory proteins of 7–11 kDa belonging to the FXYD family (Figure 2A; Blanco and Mercer, 1998; Blanco, 2005; Geering, 2006; Toyoshima et al., 2011). Furthermore, Na,K-ATPase serves as a “docking station” for multiple other proteins of those other ion transporters, receptors, cytoskeletal proteins and members of signaling proteins are known (Reinhard et al., 2013). Each Na,K-ATPase subunit type contains cysteine residues. The only reduced thiol within the beta subunit is localized at the edge to the membrane surface, diving into and out of the membrane during the pumping cycle (White et al., 2009; Bibert et al., 2011). Muscle-specific FXYD subunit pospholemman (PLM) has two thiols (Bibert et al., 2011). The catalytic α subunit is the largest of the three subunits, forms the ATP binding site and binding sites for ions and transport pore, and has 23–24 thiols depending on the isoform (Bogdanova et al., 2006). Several of these thiols are considered to be the targets of regulatory reversible thiol modifications. These modifications make the Na,K-ATPase function extremely sensitive to the changes in redox state and oxygen availability (chronic and acute hypoxia).

#### Irreversible oxidation of thiols

Oxidative stress represents an imbalance between an augmented production of pro-oxidative equivalents (mostly ROS and RNS) and/or their detoxification rate by the so-called antioxidant systems (Sies, 2015). Under these circumstances oxidants attack reduced protein thiols turning them into sulfinic or sulfonic acids. Irreversible oxidation induced in the Na,K-ATPase isolated from kidneys by its exposure to 20 mM H_2_O_2_ results in a decrease in V_max_ to a half and inability to form oligomeric complexes (Kurella et al., 1999). Sensitivity to oxidation varied for the Na,K-ATPase isozymes, those from the heart and the brain (astrocytes) formed by the α2 isoform of the catalytic subunit being more susceptible for oxidation than the ubiquitously expressed α1 isozyme (Xie et al., 1995). ATP was protecting the enzyme from irreversible oxidation (Xu et al., 1997). Depletion of cytoplasmic and mitochondrial GSH or exposure of cerebellar granule cells to hyperoxia also caused suppression of the enzyme activity associated with massive free radical burst (Petrushanko et al., 2006, 2007).

**Table 1:** **Analysis of flanking amino acids' composition of selected thiols in the α1 and α2 subunits (rat sequences)**.

**Number of Cys**	**Sequence fragment**	**Substitutions α1->α2**	**Comments**
206 α1 α2	NGCKV HGCKV .^****^	N->H	Asp-> His (neutral to positive in α2)
236 α1 α2	NAFF NCFF ^**^.^**^	A->C	Cys present in α2 and missing in α 1 isoform
458 α1 α2	EVCCGS ELSCGS ^*^:.^***^	V->L C->S	Cys missing in the α2 isoform and present in α 1 Val->Leu (neutral)
513 α1 α2	DRCSS DRCST ^****^:	S->T	Neutral substitutions
551 α1 α2	GFCHL GFCQL ^***^:^*^	H->Q	His->Gln (positive to neutral in α2)
579 α1 α2	NLCFV KLCFV :^****^	N->K	Asp->Lys (neutral to positive in α2)
932 α1 α2	VICKT IICKT :^****^	V->I	Neutral exchange

*Data shown in the table are obtained as a result of sequence alignment (Clustal O(1.2.1)) for the Na,K-ATPase α1(P06685) and α2 (P06686) subunits' sequences for rat (Rattus norvegicus), published in UniProt-Knowledgebase (Swiss-Prot collection of sequences, http://www.uniprot.org/uniprot). Included into the table are the amino acids flanking the cysteine residues for which the isoform-specific differences (substitution, omission) have been observed. Asterisks stand for the conserved amino acids whereas dots and semicolon indicate the substitutions. Cysteines are color-coded in blue and the substituted amino acids in yellow*.

As reviewed below terminal irreversible oxidation of the enzyme has two more consequences. It makes the regulatory thiols inaccessible for reversible thiol modifications and therefore renders the enzyme insensitive to redox changes and alterations in oxygen levels (Petrushanko et al., 2012). Irreversible oxidation primes the proteins to degradation (Thevenod and Friedmann, 1999). In the lungs severe hypoxia (1.5 kPa) promotes ubiquitination and lysosomal degradation of the alpha1-isozyme of the Na,K-ATPase by phosphorylation of the catalytic subunit at Ser18 by PKCzeta (redox-sensitive kinase containing cysteine clusters; Dada et al., 2003, 2007; Dada and Sznajder, 2003).

#### S-nitrosylation of thiols

S-nitrosylation (and S-nitrosation, for nomenclature see Heinrich et al., 2013) is a common thiol modification in biological systems (Martinez-Ruiz and Lamas, 2004; Martinez-Ruiz et al., 2013). Nitric oxide is unable to directly interact with reduced thiols. Several mechanisms have been described for nitrosothiol formation, which do not require the presence of specific enzymes that catalyze these reactions (Martinez-Ruiz et al., 2013).

*Direct reaction of NO to nitrosylate a cysteine thiyl radical* (P-S^•^) whenever the latter has been formed.

(1) $$\begin{array}{c} {\text{P-S}}^{•}\;+{\;}^{•}\text{NO }\to \text{ P-S-NO} \end{array}$$

This is a fast reaction may compete with NO binding to the heme of soluble guanylate cyclase sGC heme (Madej et al., 2008; reviewed in Smith and Marletta, 2012). However, its biological significance is limited to those proteins in which the thiyl radical could be formed when NO is being produced.

*NO autooxidation* can also lead to S-nitrosylation, via the formation of N_2_O_3_:

(2) $$\begin{array}{rc} 2\text{ NO}+ & {\text{O}}_{2}\;\to 2\text{ N}{\text{O}}_{2} \end{array}$$

(3) $$\begin{array}{rc} \text{N}{\text{O}}_{2}+ & \text{NO}\to {\text{N}}_{2}{\text{O}}_{3} \end{array}$$

(4) $$\begin{array}{rc} {\text{N}}_{2}{\text{O}}_{3}+ & \text{RSH}\to \text{RSNO}+{\text{H}}^{+}+\text{N}{\text{O}}_{2}^{-} \end{array}$$

The rate-limiting reaction is the formation of NO_2_, so it requires a very high NO concentration for producing a significant amount of S-nitrosylation. This high NO concentration can be achieved in the proximity of NO sources, mainly NOS. Indeed, the reaction is 30-fold faster in hydrophobic environments (Moller et al., 2007), suggestion that it can be favored in regions close to cell membranes, where NOS enzymes localize.

Transnitrosylation in reactions involving modified cysteine residues, including S-nitrosoglutathione, has emerged as an effective and regulated mechanism forming this modifications (Nakamura and Lipton, 2013; Kohr et al., 2014):

(5) $$\begin{array}{l} \text{RSNO}+\text{R}’\text{SH}\to \text{RSH}+\text{R}’\text{SNO }(\text{trans }-\text{ nitrosylation},\\ \text{ nitrosylated glutathione may be involved}) \end{array}$$

Interaction of peroxynitrite with cysteine thiol residues does not give S-nitrosylated products directly but causes oxidation of thiols to sulfenic acid that in turn can form mixed disulfides, in particular with glutathione (see S-glutathionylation below; Alvarez and Radi, 2003):

(6) $$\begin{array}{l} {\text{RS}}^{-}+\text{ONOOH }\to \text{RSOH}+{\text{NO}}_{2}^{-}\\ {\text{RSO}}^{-}+\text{GSH}\to \text{RSSG}+{\text{OH}}^{-} \end{array}$$

Denitrosylation mainly involves two mechanisms (Smith and Marletta, 2012). GSNO concentration is controlled by enzymatically-catalyzed NADH (for GSNO reductase (GSNOR) or NADPH-dependent (carbonyl reductase 1, CBR1) reduction to GSH. Since trans-nitrosylation of thiols involves GSNO (reaction 5), this process may be considered as rate-limiting in S-nitrosylation of proteins. This is true for the Na,K-ATPase, as increase in the levels of GSNO induces the second trans-nitrosylation step and formation of S-glutathionylated adducts of the α (Li et al., 2001; Petrushanko et al., 2015) and β (Liu et al., 2013) subunits of the enzyme. Thioredoxin catalyzes denitrosylation of multiple targets by trans-nitrosylation of its active cysteines Cys32 and 35.

Hypoxia alters the number of S-nitrosylated cysteines in numerous proteins. In endothelial cells with high levels of eNOS (NOSIII) multiple targets get S-nitrosylated under hypoxic conditions. This NO synthase has high affinity to O_2_ (Kd 0.29 kPa, Dweik, 2005) and can therefore support NO production at low O_2_ levels. At the same time in heart tissue, where NOSI and NOSII with low O_2_ affinity are dominating (Kd 23–35 kPa, Dweik, 2005) hypoxia is associated with rapid cessation of NO production and decrease in S-nitrosylation of cysteine residues of the α subunit of the Na,K-ATPase (Yakushev et al., 2012). NO and S-nitrosylation were shown to protect thiols from irreversible oxidation and S-glutathionylation, and allow the enzyme to maintain high activity in the brain and heart (Petrushanko et al., 2007; Yakushev et al., 2012). Irreversible oxidation makes regulatory thiols inaccessible for S-nitrosylation. This may explain the fact that the sensitivity of the Na,K-ATPase to NO was lost in a mouse model of amyotrophic lateral sclerosis (Ellis et al., 2003).

#### S-glutathionylation of thiols: Chemistry

S-glutathionylation (also referred to as S-glutathiolation), the formation of a mixed disulfide between a protein cysteine and that of glutathione. This thiol modification is produced in a number of chemical reactions (Klatt and Lamas, 2000; Dalle-Donne et al., 2007, 2008; Martinez-Ruiz and Lamas, 2007; Mieyal et al., 2008; Bachi et al., 2013).

Reactions resulting in formation of S-glutathionylated protein adducts include classical thiol disulfide exchange (reaction 7) that does not require any catalysis, and several mechanisms requiring prior oxidative modification of the thiol residues (either the protein thiol or cysteine residue of GSH, reactions 8–13).

(7) $$\begin{array}{r} \text{P-}{\text{S}}^{-}+\text{GSSG}\to \text{P-SSG}+\text{G}{\text{S}}^{-} \end{array}$$

Reactions priming thiols to S-glutathionylation include prior formation of thiyl radicals with O_2_ as an electron acceptor (reactions 8–9), oxidation of thiols to sulphenic acid (reaction 10–11) or to nitrosothiol (reactions 12–13)

(8) $$\begin{array}{rcl} \text{P}-{\text{S}}^{•} & + & \text{G}{\text{S}}^{-}+{\text{O}}_{2}\to \text{P}-\text{SSG}+{\text{O}}_{2}^{•-} \end{array}$$

(9) $$\begin{array}{ll} \text{P}-{\text{S}}^{-}+ & \text{G}{\text{S}}^{•}+{\text{O}}_{2}\to \text{P}-\text{SSG}+{\text{O}}_{2}^{•-}\\ \left(\text{catalyzed by glutaredoxin}\right) \end{array}$$

(10) $$\begin{array}{rc} \text{P-SOH}+ & \text{GSH}\to \text{P-SSG}+{\text{H}}_{2}\text{O} \end{array}$$

(11) $$\begin{array}{rc} \text{P-SH}+ & \text{GSOH}\to \text{P-SSG}+{\text{H}}_{2}\text{O} \end{array}$$

(12) $$\begin{array}{rc} \text{P-SNO}+ & \text{GSH}\to \text{P-SSG}+\text{HNO} \end{array}$$

(13) $$\begin{array}{rc} \text{P-SH}+ & \text{GSNO}\to \text{P-SSG}+\text{HNO} \end{array}$$

S-glutathionylation of thiols in hypoxic cells is triggered by a local shift in GSSG/GSH ratio toward more GSSG (Petrushanko et al., 2012; Yakushev et al., 2012). On the other hand, oxidative stress following superoxide anion burst in hypoxic mitochondria, will promote oxidation of thiols to sulfenic acid priming them to S-glutathionylation via reaction 10.

Reversibility of S-glutathionylation is supported. Glutaredoxin1 and 2 may catalyze de-glutathionylation or glutathionylation reactions depending on the redox environment (NAD(P)H:NAD(P), GSH:GSSG, NO availability; Mieyal et al., 2008). Not all thiols in the proteins are accessible for S-glutathionylation. Whereas glutaredoxin1 is active in the cytosol and inter-membrane space, whereas glutaredoxin 2 is localized in the mitochondrial matrix, controlling the state of protein thiols in these compartments (Allen and Mieyal, 2012). Further enzymes capable to catalyze de-glutathionylation reaction include sulfiredoxin (Lei et al., 2008), glutathione transferase P (Townsend et al., 2009), and glutathione transferase omega 1 (Menon and Board, 2013).

Not all thiols are equally accessible for S-glutathionylation. Specific conditions that have to be fulfilled for the thiol to become glutathionylated are discussed below.

#### Localization of the groups favoring glutathionylation

The likelihood for a given thiol to undergo S-glutathionylation is defined by several factors: (i) dissociation constant pKa of a thiol (ii) microenvironment (amino acid composition in vicinity to the cysteine residue), (iii) accessibility of the group and steric restrictions, and (iv) redox potential of a thiol (Nagy, 2013). Nucleophilic substitution reaction in which S-glutathionylated adduct is formed involves interaction of a thiolate anion with a GSSG molecule (Allen and Mieyal, 2012). Thiol therefore has to be deprotonated to join this reaction. The average pKa of majority of cysteine residues is 8.5. Thus, probability of deprotonation of cysteine residues under physiological conditions is relatively low making S-glutathionylation selective (Dalle-Donne et al., 2009). Reduction in pKa of distinct thiols is supported by the positive charge of three flanking amino acids such as arginine, lysine, or histidine (Allen and Mieyal, 2012; Sun et al., 2013; Zhao et al., 2015). Proximity of negatively charged amino acids to a thiol on the contrary compromises interaction with GSSG. Protein sequence analysis for the α1 subunit of the Na,K-ATPase revealed that cysteines 204, 452, 599, and 698 (corresponding to the Cys 206, 454, 601, and 700 in Figure 2) are prone to S-glutathionylation (Mitkevich et al., 2016).

Steric restriction and localization of cysteine residue within the protein sequence are yet other parameters that should be taken into consideration when predicting S-glutathionylation sites (Pineda-Molina et al., 2001). Hydrophilicity of GSSG suggests that the residues should be exposed to aqueous phase as the reaction with GSSG occurs. Furthermore, the exposed surface of thiol should be sufficient for docking of GSSG to form mixed dithiols (Sun et al., 2013). “Basal S-glutathionylation” of cysteines that are buried within the protein, but are S-glutathionylated, represents a special case and is discussed below.

Further restrictions limiting the number of S-glutathionylated residues are related to the redox state of a thiol. For most proteins GSH:GSSG ratio should decrease from 100:1 to 1:1 to achieve S-glutathionylation of 50% of thiols (Allen and Mieyal, 2012). Very few proteins, such as c-Jun may be glutathionylated within physiological range of half-cell redox potential for GSH:GSSG couple (Klatt et al., 1999; Allen and Mieyal, 2012). Na,K-ATPase is one of these few proteins as interaction of the cysteine residues within its catalytic α subunit with GSSG can be observed as GSSG reaches 150 μM levels in the presence of 1.5 mM GSH (Petrushanko et al., 2012; Yakushev et al., 2012).

### S-glutathionylation and targets in the Na,K-ATPase

#### Alpha subunit of the enzyme

Cysteines of all three types of subunits forming Na,K-ATPase undergo S-glutathionylation. Catalytic α subunit contains 23 or more (depending on the isoform) cysteine residues (Figure 2A). Fifteen out of 23 Cys residues of the α1 isoform are localized within the cytosolic loops and are easily accessible for interaction with cytosolic GSSG. Out of seven cysteine residues of the regulatory β subunit 6 are forming S-S bonds with each other and only one remaining cysteine possesses a thiol accessible for S-glutathionylation (White et al., 2009). Tissue-specific FXYD subunits also contain 1–2 cysteines of which at least one undergoes S-glutathionylation (Bibert et al., 2011).

*Regulatory S-glutathionylation* Cysteine residues that were found S-glutathionylated within the α1 subunit isolated from duck salt gland were classified as targets for regulatory or basal S-glutathionylation (Petrushanko et al., 2012). Whereas basal S-glutathionylation is not associated with the changes in the enzyme hydrolytic activity, binding of glutathione to the regulatory cysteine residues causes the enzyme's complete inactivation that can be reversed upon de-glutathionylation (Petrushanko et al., 2012; Yakushev et al., 2012). Regulatory S-glutathionylation could be accomplished by incubation of the purified enzyme or membrane fraction with GSSG indicating that regulatory thiols were accessible for classical thiol disulfide exchange (reaction 5). Similar response was obtained for the α1 subunit within the broad range of tissues and cell type (cell line, salt gland, kidneys, heart tissue) and species (duck, rabbit, pig, mouse, rat, Spalax mole rat, trout, and human; Petrushanko et al., 2012, 2015; Yakushev et al., 2012; Juel, 2014; Xianyu et al., 2014; Juel et al., 2015; Mitkevich et al., 2016). Apart of α1, S-glutathionylation of the α2 isozyme was shown in heart and skeletal muscle. Moreover, α2 isozyme appeared to be more sensitive to GSSG-inducible inhibition in rat heart (Petrushanko et al., 2012; Xianyu et al., 2014; Juel, 2016). This observation is in agreement with the report of Xie on exceptionally high susceptibility of the α2 subunit to oxidation (Xie et al., 1995) and suggests that Cys residue(s) within this isoform are more accessible for oxidation (e.g., Cys 579 in Table 1). As can be seen from the Table 1, microenvironment for two cysteine residues (Cys 206 and 579) within the cytosolic loops of the α2 subunit favors S-glutathionylation as non-charged amino acids present in the α1 isoform are substituted by positively charged ones facilitating thereby deprotonation of the thiols. Furthermore, α2 subunit harbors the extra cysteine (Cys 236) in the actuator domain and lacks the Cys458 (present in the α1). Thus, although the number of cysteines does not differ between the α1 and α2 subunits, their location, pK and the ability to become S-glutathionylated show clear isoform-specificity.

Localization of the sites of regulatory S-glutathionylation was identified by means of mass spectrometry (Petrushanko et al., 2012). Three of them, Cys 454, 456, and 459 are proximal to the ATP binding site whereas Cys 244 is localized in the small cytosolic loop that may approach the ATP binding pocket in E2 conformation (Bogdanova et al., 2006). Binding of glutathione to the regulatory sites displaces adenine nucleotides from interaction with the enzyme (Petrushanko et al., 2012). In turn, GSSG cannot block the enzyme's hydrolytic activity in the presence of ATP or ADP (Petrushanko et al., 2012; Xianyu et al., 2014). ATP was showing maximal “protective effect” compared to the other nucleotides (Xianyu et al., 2014). Interaction with ATP (but not with the other nucleotides) brings the nucleotide-binding domain of the α subunit closer to the phosphorylation domain (E1-closed state) shielding thiols of the small and large cytosolic loops from attack by GSSG or oxidants (Petrushanko et al., 2014) and making them less prone to irreversible oxidation (Xie et al., 1995; Xu et al., 1997).

Increase in S-glutathionylation of the α subunit was triggered by hypoxia associated with mild oxidative stress and modest ATP depletion in rat heart and SC1 cell line derived from mouse fibroblasts (Petrushanko et al., 2012, 2015; Yakushev et al., 2012). Inhibition of the Na,K-ATPase and induction of S-glutathionylation could be mimicked by the modulation of intracellular redox state by exposure of cells to glutathione derivatives et-GSH, GSNO, GSSG, or depletion of the intracellular GSH (Petrushanko et al., 2006, 2015). S-glutathionylation of the α subunit cannot be sustained under anoxic conditions. This thiol modification is induced by 0.2% O_2_ but less pronounced at 0.05% O_2_ in mouse fibroblast-derived cell line (Petrushanko et al., 2015).

##### Basal S-glutationylation of the α subunit

Basal S-glutathionylation of the α subunit of the Na,K-ATPase is not associated with the changes in the enzyme function. 15 cysteine residues facing the cytosol are accessible for S-glutathionylation. Treatment of the cell lysates, membrane fractions or purified active Na,K-ATPase preparations with reducing agents (e.g., by Dithiotreitol or Tris(2-carboethyl)-phosphine, TCEP) could not completely remove glutathione bound to the α subunit's thiols (Mitkevich et al., 2016). Complete de-glutathionylation could only be achieved under conditions supporting partial denaturation of the protein (8 M urea and 8% SDS). De-glutathionylation of the cysteines inaccessible for reducing agents in the protein retaining its native structure was associated with substantial loss of the enzyme activity due to unfolding. The only chance these Cys residues shielded from GSSG and reducing agents have to acquire S-glutathionylation is before the folding was completed (Figure 2B). This implies that S-glutathionylation of certain Cys residues is a co-translational rather than post-translational modification and it may be required for correct protein folding. Detailed analysis of the X-ray structures of the porcine α1 subunit-containing enzyme (PBD codes: 3B8E, 3KDP, 3WGU, 3WGV, 4HYT) revealed a number of isolated cavities with unresolved electron density next to the Cys residues (numbering as in Figure 2B) Cys 206–Cys 244; Cys 454–Cys 458–Cys 459; Cys 700–Cys 369, Cys 601 (Mitkevich et al., 2016).

These regions of relatively high residual electron density that cannot be explained by the presence of water are sufficient in size to home glutathione. However, so far no X-ray structures of the actual catalytic subunit with S-glutathionylated cysteine adduct was reported. Detection of glutathionylated residues in crystal structures of proteins is not impossible (Srinivasan et al., 2014), but much less common that detection of these modifications by means of mass spectrometry. One of the technical approaches to use for identification of S-glutathionylated cysteine residues is tracking for glutathione localisation using analysis of unresolved density next to the cysteine residues. This approach was used for identification of glutathione bound to the ABC transporter Atm1 (Srinivasan et al., 2014). In each cavity of the α subunit only one Cys residue was reported to be S-glutathionylated (basal type of S-glutathionylation, Figure 2B, Mitkevich et al., 2016). These findings imply that Cys454 is located in a cavity and, hence, cannot be a regulatory thiol as suggested earlier (Petrushanko et al., 2012). Its basal S-glutathionylation will contribute to the protein folding instead. Out of the Cys206-Cys244 couple Cys 206 is capable of basal S-glutathionylation whereas Cys 244 carries a regulatory thiol group. In contrast to regulatory S-glutathionylation observed within minutes after the drop in O_2_ availability, increase in basal S-glutathionylation is only observed after 72 h of hypoxic exposure (Mitkevich et al., 2016). This kinetics correlate with the onset of *de novo* protein synthesis rather than acute alterations in thiol state.

Moreover, as this modification is retained during the life-span of a protein it represents “redox memory” that reflects the cellular redox state at the moment of synthesis of this molecule. Thus, this “redox memory” may represent the process of adaptation to the alterations in redox state occurring in particular in response to deoxygenation. The fact that basal S-glutathionylation levels differ in muscle fibers being higher in oxidative fibers compared to the glycolytic fiber type suggests that it is likely to be associated with the metabolic state of the tissue (Juel, 2014).

#### S-glutathionylation of beta subunit

A single reduced thiol group in β subunit of the Na,K-ATPase, Cys 46 (Figure 2A), is the one that may undergo reversible post-translational modifications. S-glutathionylation of this cysteine residue was reported for all three muscle types (skeletal, smooth, and heart muscles; Figtree et al., 2009; Liu et al., 2013; Juel et al., 2015). Since interaction of glutathione with Cys 46 results in down-regulation of the enzyme function this residue is a site of regulatory S-glutathionylation (Figtree et al., 2009). Inhibitory effect is achieved due to the weakening of the interaction between α and β subunits upon S-glutathionylation (Figtree et al., 2009). S-glutathionylation of β subunit does not inactivate the enzyme completely (Figtree et al., 2009). Unfortunately, the studies in which S-glutathionylation of the β subunit was detected were not presenting the information on the degree of S-glutathionylation of the α subunit, making it impossible to discriminate between the impacts of these two processes into the enzyme activity regulation (Liu et al., 2013).

In contrast to the regulatory cysteines of the catalytic α subunit that are readily interacting with GSSG Cys 46 does not join reaction of classical thiol disulfide exchange with GSSG (Petrushanko et al., 2012). This may be attributed to the localization of this Cys residue. According to the X-ray structure it is buried within the membrane in E2 2K+Pi conformation (Ogawa et al., 2009) and is only accessible for S-glutathionylation in E1ATP and E1Na(3) conformation (Liu et al., 2012). Furthermore, Cys 46 may become accessible or S-glutathionylation upon the loss of association between the α and β subunits (Garcia et al., 2015). Ouabain stabilizes the enzyme in E2 conformation and compromises S-glutathionylation of β subunit (Liu et al., 2012). De-glutathionylation can be achieved by treatment of the S-glutathionylated enzyme with Glutaredoxin.

Localization of the regulatory cysteine defines conditions required for its S-glutathionylation. It only occurs in the presence of peroxynitrite and hence involves reactions 12 and 13. Physiological and pathophysiological role of this form of regulatory thiol modification in the heart has been intensively investigated. Stimulation of ${\text{O}}_{2}^{•-}$ production by NADPH oxidases, that co-localize and co-immunoprecipitate with the Na,K-ATPase, induces S-glutathionylation of the β subunit at Cys46 (Liu et al., 2013). The stimulation of NADPH oxidases could be triggered by their phosphorylation by PKC produced upon activation of β1/β2 adrenoceptors or treatment of the myocardium with angiotensin II. Furthermore, increase in S-glutathionylation of the β subunit was reported in the infarcted area in sheep heart (Figtree et al., 2009). Similar effect was achieved by exposure of cardiomyocytes to the activator of adenylate cyclase forskolin and the following activation of PKCϵ (White et al., 2010) or direct administration of ONOO- (Figtree et al., 2009). Scavenging of ${\text{O}}_{2}^{•-}$ using superoxide dismutase on the contrary abolishes S-glutathionylation of the β subunit. Similar effect may be achieved by the stimulation of sGC that interfered with phosphorylation and activation of NADPH oxidases (Chia et al., 2015). Activation of β3 adrenoreceptor is a physiological stimulus decreasing S-glutathionylation of the Cys 46 (Bundgaard et al., 2010). These differential responses are easily explained based on the chemistry of S-glutathionylation of thiols. Deoxygenation is associated with suppression in production of NO by NOS1 and NOS2 as well as in reduction of activity of NADPH oxidases Kd of which for O_2_ is within 2 kPa range. Thus, decrease in O_2_ levels below this threshold reduces production of peroxynitrite and decreases S-glutathionylation of Cys 46 in β subunit. Uncoupling of oxidative phosphorylation and ${\text{O}}_{2}^{•-}$ burst in the mitochondria on the contrary will facilitate GSSG production in the cytosol and induce S-glutathionylation of regulatory cysteines within the cytosolic loops of the catalytic α subunit.

#### S-glutathionylation of FXYD subunits

Tissue-specific FXYD proteins associate with Na,K-ATPase αβ complex stabilizing it and modulating the enzyme activity (Geering, 2006). These modulatory subunits also contain 1 or 2 cysteine residues that undergo reversible thiol modifications. Most of the information on the role of these modifications in control of Na,K-ATPase function was obtained for the cardiac-specific FXYD1 subunit also known as phospholemman (PLM). It contains 2 Cys residues (C1 corresponding to Cys40 and C2 corresponding to Cys42 in human FXYD1 protein, Figure 2A), and S-glutathionylation of the C2 was shown to correlate reciprocally with the availability of glutathionylated form of the β subunit (Bibert et al., 2011). Localization of C2 and mechanisms of induction of S-glutathionylation of PLM are most likely shared with the β subunit of the Na,K-ATPase and involve peroxynitrite. Interaction of PLM with glutathione may be triggered by forskolin and prevented by exposure to superoxide dismutase. De-glutathionylation is also catalyzed by glutaredoxin1 or treatment with DTT. Thus, both PLM and β subunits lack cysteines that are not accessible for de-glutathionylation (redox memory). Physiological stimuli increasing the level of PLM S-glutathionylation include angiotensin II and infarction (Bibert et al., 2011).

The importance of the amino acids flanking the cysteine residue in control of accessibility of it for S-glutathionylation was emphasized as the amount of glutathionylated adducts was compared for several members of FXYD family. C2 cysteines were found to be prone to S-glutathionylation in FXYD2 (renal) and FXYD7 (brain-derived) (Geering, 2006; Bibert et al., 2011). Phospholemman (PLM, FXYD1) interacts with both catalytic and regulatory subunits of the Na,K-ATPase. Thus, interaction between α and β subunits and the enzyme function is affected by S-glutathionylation of the FXYD1. Furthermore, the reciprocal regulation of S-glutathionylation of the β and FXYD subunits suggests that FXYD may reverse the inhibitory action mediated by binding of glutathione to the regulatory β subunit on the Na,K-ATPase. It remains unclear if glutathione is transferred from the Cys46 on to the β subunit to the C2 residue in reaction of thiol disulfide exchange of the FXYD protein or if S-glutathionylation of the FXYD protein makes Cys46 inaccessible for S-glutathionylation.

### H_2_S and the Na,K-ATPase

Hypoxic exposure was shown to cause a rapid increase in H_2_S levels in a number of cells including chemoreceptory glomus cells (Peng et al., 2010; Olson, 2015; Yuan et al., 2015). Direct interaction of H_2_S with the Na,K-ATPase has not been reported. However, exposure of renal tubular epithelial cells to H_2_S causes internalization of the Na,K-ATPase in complex with other proteins. This inhibitory response of the pump is triggered by sulfhydrolation of cysteines 797 and 798 at the epidermal growth factor receptos (EGFRs) (Ge et al., 2014).

## Coordinated regulation of redox-sensitive protein networks by reversible thiol modifications

S-glutathionylation and S-nitrosylation are altering the function of numerous proteins that form redox sensitive networks (Sun et al., 2006; Dalle-Donne et al., 2009). Protein clusters forming these networks are the ones controlling metabolism, ion transport, cell fate and cycle, adaptation, and others. This type of free radical-based signaling is of key importance in responses to hypoxia and reoxygenation mediating acute and long-term tissue-specific coordination of function of multiple proteins. S-glutathionylation of the α subunit of the Na,K-ATPase occurs among with alterations in thiol state of other proteins. The most obvious protein partners that undergo S-glutathionylation are the adjacent β and FXYD subunits. Thiol disulfide exchange in which PLM most likely acquires glutathione from the Cys 46 subunit of the β subunit is suggested to cause dissociation of the FXYD subunit of from the Na,K-ATPase as it does not co-precipitate with the α subunit (Bibert et al., 2011). As PLM is a regulatory subunit that is shared between the Na,K-ATPase and the Na/Ca exchanger, it was suggested that S-glutathionylation as well as its redox-sensitive phosphorylation of it impacts activity of both transporters (Silverman et al., 2005; Cheung et al., 2007; Zhang et al., 2011). Calcium handling in the heart is intimately linked to the Na^+^ levels (Shattock et al., 2015) and, hence to the activity of the α2 isozyme of the Na,K-ATPase that is particularly prone to inhibition by GSSG (James et al., 1999; Petrushanko et al., 2012). Apart of the Na/Ca exchanger reversible thiol modifications are known to control the activity of L-type Ca^2+^ channel, RyR2 ryanodine receptors, SERCA2a Ca^2+^ pump and multiple other ion transporters (Sun et al., 2006; Bull et al., 2008; Lancel et al., 2009; Donoso et al., 2011).

These coordinated redox-driven changes in activity of multiple ion transporters may support cytoprotection and survival of the cell/tissue/organism or promote death under hypoxic conditions (see Section Regulation of the Na,K-ATPase Activity by Cardiotonic Steroids).

## Oxygen-sensitive phosphorylation

**Figure 3:**
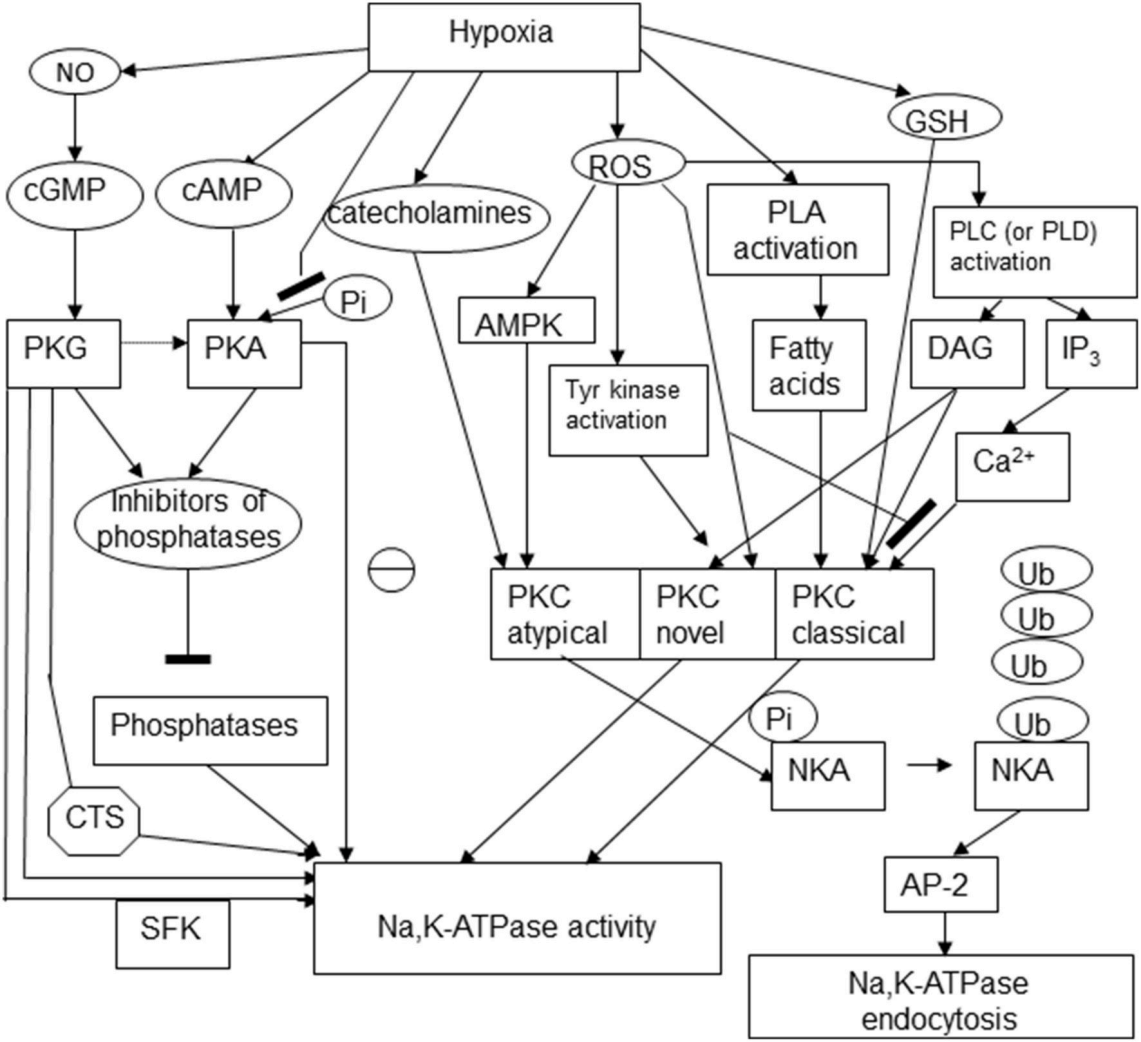
**Schematic representation of the multiple pathways involved in regulatory phosphorylation of the Na,K-ATPase under hypoxic conditions**. The following abbreviations were used in the scheme: cyclic adenosine and guanosine monophosphates (cGMP and cAMP), protein kinases C, A, and G (PKC, PKA, PKG), cardiotonic steroids (CTS), Src kinase family (SFK), AMP-activated protein kinase (AMPK), phospholipase C (PLC), diacylglycerol (DAG), inositol 3-phosphate (IP3), ubiquitin (Ub), NKA (Na,K-ATPase), adaptor protein 2 (AP-2).

Regulatory phosphorylation is known to control the activity, sensitivity to the cardiotonic steroids, and availability of the Na,K-ATPase on the plasma membrane. Changes in phosphorylation state of the enzyme were reported in response to hypoxic challenge and for a long time believed to be the only mechanism involved in “channel arrest” response of the Na,K-ATPase in hibernating animals (MacDonald and Storey, 1999; Ramnanan and Storey, 2006; McMullen and Storey, 2008). At present “channel arrest” responses seem to be more versatile, and yet triggered by a single mechanism of regulation, in this case not only the Na,K-ATPase itself, but also the redox-sensitive kinases, namely, reversible thiol modifications of regulatory cysteine residues or action of gasotransmitters. Phosphorylation sites at the α, β and FXYD subunits are schematically presented in Figure 3.

### Protein kinase C (PKC)

Hypoxic exposure of alveolar epithelial cells was shown to trigger actively controlled internalization of the Na,K-ATPase in the form of clathrin-coated vesicles (Dada et al., 2003). This process was induced by phosphorylation of the α subunit of the Na,K-ATPase by at atypical PKC isozyme.

Novel and classical PKC isoforms are also capable to alter activity of the Na,K-ATPase in various cell types. Classical (conventional α, β1 and β2, and γ isoforms) and novel isoforms PKC δ, ε, η, θ, and μ may be activated diacylglycerol (DAG), phorbol esthers and phosphatidylserine and are sensitive to the changes in Ca^2+^ in the cell. Atypical PKCι and ξ retain the sensitivity to phosphatidylserine but are Ca^2+^-insensitive (Figure 3; Newton, 1995; Parker and Murray-Rust, 2004).

Catalytic core of PKC contains critical cysteine-rich motifs that are conserved in all PKC isozymes making all proteins of this class redox-sensitive (Newton, 1995). This motif is duplicated in classical and novel PKC isoforms, whereas in atypical PKCs only one repeat is found. Oxidation of these cysteines causes transient activation of the enzyme but makes it unable to interact with DAG and phorbol esters making it Ca^2+^- and DAG-insensitive (Gopalakrishna and Anderson, 1989). S-glutathionylation of these regulatory thiols is inactivating α, βI, βII, γ, δ, ε, and ζ isoforms of PKC. Formation of glutathionylated adducts occurs within physiological range of GSH concentrations (0.5–10 mM) and results in the enzyme inactivation (Ward et al., 1998, 2000; Chu et al., 2001). In addition, PKC may undergo S-nitrosylation that was shown to suppress activity of PKCα (Choi et al., 2011). Depletion of intracellular GSH by exposure to conjugating agent diethylmaleimide or inhibition of *de novo* GSH production by L-buthionine-S,R-sulfoximine results in reduction in activity of conventional PKC isoforms (α, βI, and βII) by 35–50%, along activation of the novel isoforms (δ and ε) (Domenicotti et al., 2000). This response of the PKCs to the changes in redox state will induce endocytosis of the Na,K-ATPase (Figure 3).

PKC phosphorylates the catalytic α1 subunit at Ser11 and 18 (rat sequence) at the N-terminus of the protein that forms the actuator domain. Ser18 that is considered as a major phosphorylation site is available for phosphorylation in the E2 conformation (Feschenko and Sweadner, 1997). In the E1 conformation the N-terminus is translocated approaching the small cytosolic loop that shields the phosphorylation site from interaction with the kinase (Feschenko et al., 1997; Segall et al., 2003). The analogs of Ser18 and Ser11 are missing in the α2 isoform of the catalytic subunit (compare sequences A24639 for α1 and B24639 for α2 subunit in PubMed protein sequence library http://www.ncbi.nlm.nih.gov) making this isozyme PKC-insensitive. Phosphorylation efficiency may also vary depending on the PKC isoform. Conventional isoforms are more efficient in phosphorylation of the α1 isoform of the pump isolated from rat retinal cells than the novel isoforms δ and ε (Kazanietz et al., 2001). Phosphorylation of the α subunit by PKC may have stimulatory of inhibitory effects on the Na,K-ATPase depending on the cell type (Therien and Blostein, 2000). It is suggested that phosphorylation modulates interaction of the enzyme with other proteins or trigger its internalization (Feschenko and Sweadner, 1997). For example, activation of the atypical isoforms of PKC ξ triggers internalization of the Na,K-ATPase in alveolar epithelial cells under hypoxic conditions (Dada and Sznajder, 2003). This effect is initiated by the free radical burst in the mitochondria that is followed by the activation by the 5′-AMP-activated protein kinase (AMPK) that is phosphorylated at Thr172. The activated AMPK phosphorylates PKCξ at Thr410 and its translocation to the plasma membrane (Gusarova et al., 2009). Upon translocation PKC phosphorylates Na,K-ATPase at the Ser18 residue in the N-terminus of the α1 subunit. Endocytosis of the α1-containing isozyme of the ATPase is precluded by ubiquitination of the lysine residues next to the Ser18 (Dada et al., 2007; Lecuona et al., 2007). Ubiquitination makes the enzyme recognizable for the mu2 subunit of the adaptor protein that binds to the Tyr527 of the α1 subunit initiating its endocytosis in clathrin-coated vesicle is initiated with the following degradation of the Na,K-ATPase (Chen et al., 2006; Lecuona et al., 2007).

PLM is one more target of phosphorylation by PKCϵ at Ser36 and Ser 68. Phosphorylation it stimulated by Ca^2+^ and is NO-dependent (Pavlovic et al., 2013). Ischemia was associated with facilitation of phosphorylation of PLM, its de-attachment from the αβ complex and increase in the Na,K-ATPase hydrolytic activity in sarcoplasmic membrane fraction (Fuller et al., 2003, 2009).

### Protein kinase G (PKG)

Hypoxia and reoxygenation result in the alterations in NO, CO, and H_2_S production (see above). All gasotransmitters are capable of activation of sGC that in turn triggers activation of cGMP-dependent protein kinase PKC (PKG) (Therien and Blostein, 2000; Chen et al., 2015). Similar to that for PKC, phosphorylation by PKG was reported to have diverse effects on the Na,K-ATPase activity (Therien and Blostein, 2000). It remains unclear if PKG can directly access the phosphorylation site within the α subunit, as it could only be phosphorylated in the presence of detergents in the purified protein preparation (Fotis et al., 1999; Beltowski et al., 2003). However, treatment of yolk-free homogenates of Xenopus oocytes with cGMP results in phosphorylation and activation of the Na,K-ATPase in the absence of detergents (Fotis et al., 1999). It may reflect the indirect action of PKG on the ATPase via the suppression of dephosphorylation. Yet one more report on the activation of the α2/3 isozymes of the Na,K-ATPase in the central nervous system involves activation of cGMP-PKG pathway following the stimulation with glutamate (Munhoz et al., 2005; Scavone et al., 2005). On the other hand, supplementation of NO donors was shown to inhibit the Na,K-ATPase in nonpigmented epithelial cells of porcine eyes that is associated with activation of PKG (Shahidullah and Delamere, 2006). This effect involves PKG-driven activation of src-family kinases (Shahidullah et al., 2014).

Apart of the alterations in the enzyme activity, stimulation of the cGMP-PKG pathway may affect binding of cardiotonic steroids to the Na,K-ATPase. Increases the sensitivity of the renotubular Na,K-ATPase to marinobufagenin triggered by atrial natriuretic peptide is mediated by the cGMP-PKG signaling cascade (Fedorova et al., 2012).

### Protein kinase A (PKA)

Cyclic AMP-sensitive protein kinase (PKA) is a redox-sensitive enzyme containing regulatory thiols (Brennan et al., 2006). In the absence of cAMP the enzyme exists in the inactive tetrameric state and the regulatory Cys199 within the C subunits is inaccessible for regulatory S-glutathionylation (Humphries et al., 2002). Binding of glutathione to this cysteine residue inactivates the kinase and enhances dephosphorylation (Humphries et al., 2005). Vector of the changes in activity of PKA in response to deoxygenation is very much dependent on the cell type and on the activity of G-protein coupled receptors signaling via cAMP-PKA transduction pathway (Jiang et al., 2011).

PKA phosphorylates the catalytic α subunit at Ser938 (rat α1 sequence) within the cytosolic M8-M9 loop. This loop may interact with the M10, C-terminus and the third Na^+^ binding site. In line with that phosphorylation at Ser938 decreases affinity of this Na^+^ binding site to Na^+^ and thereby suppresses the enzyme function (Einholm et al., 2016).

In the purified Na,K-ATPase protein preparation this target is accessible for phosphorylation in the presence of detergent (Feschenko and Sweadner, 1994; Lutz et al., 1996). In intact COS cells phosphorylation of the α subunit at Ser943 is triggered by β-adrenergic stimulation with the following inhibition of hydrolytic and transport activity of the Na,K-ATPase (Cheng et al., 1997).

Activation of cAMP-PKA-dependent pathways in NRK-52E and L6 cell lines may suppress activity of PKC and reduce phosphorylation of the Na,K-ATPase at the PKC binding sites (Feschenko et al., 2000). This cross-talk may result from the close proximity of the PKG binding site to that of PKC (Kruger et al., 2003).

Along with PKC, PKA may catalyze phosphorylation of PLM at Ser68 (Fuller et al., 2004, 2009) in response to ischemia or β adrenergic stimulation (Despa et al., 2005) causing release of the inhibitory action of association of the FXYD1 subunit with the Na,K-ATPase.

## Regulation of the Na,K-ATPase activity by cardiotonic steroids

Cardenolides and bufadienolides are the two classes of endogenous cardiotonic steroids that serve as hormones selectively interacting with Na,K-ATPase. These compounds are produced by midbrain and adrenocortical cells, and released into the circulation in sub-micromolar concentrations in response to various stimuli such as angiotensin II, acetylcholine, vasopressin, catecholamines, and hypoxic exposure (Bagrov et al., 2009). Interaction of these very low doses of endogenous inhibitors with the Na,K-ATPase does not compromise the transmembrane Na^+^ gradients, but induces activation of Src kinase and formation of protein complex in which Src kinase and Na,K-ATPase associate with epidermal growth factor receptor (EGRF) and initiate several signaling cascades (Li and Xie, 2009). Signaling modalities depend on the type of cardiotonic steroid and its dose (Dvela et al., 2007). At the molecular level these differences in physiological action of cardenolide ouabain and bufadienolide marinobufagenin are reflected by the specific pattern of conformational changes unique for each steroid upon binding to the purified Na,K-ATPase enzyme (Klimanova et al., 2015). Affinity of the Na,K-ATPase to ouabain is maximal in E2P conformation (in this conformation binding constant for ouabain exceeds that for marinobufagenin by 17-fold), whereas marinobufagenin does not discriminate between the E1 and E2 conformations when binding to the enzyme (Klimanova et al., 2015).

Notably, substitution of Cys 244 by Ala makes cells ouabain-intolerant (Shi et al., 2000). This cysteine is a target of regulatory S-glutathionylation (Petrushanko et al., 2012). Furthermore, binding of cardiotonic steroids fixes the enzyme in distinct conformation depending on the type of cardiotonic steroids (Klimanova et al., 2015). Thus, binding of cardiotonic steroids may alter susceptibility of cysteine residues to S-glutathionylation and, *vice versa*, reversible thiol modifications may possibly alter sensitivity of the enzyme to cardiotonic steroids. This hypothesis awaits further investigations.

Local (renal) or systemic reduction in oxygen availability was shown to trigger release of cardiotonic steroids (bufadienolides or endogenous ouabain) into the circulation in rodents and humans (Zhao et al., 1995; Bagrov et al., 1998; De Angelis and Haupert, 1998; Tian et al., 2010). Apart of the induction of cytoprotective signaling pathway, endocytosis of Na,K-ATPase will support reduction of ATP consumption by hypoxic tissue (De Angelis and Haupert, 1998). Protective effect of distinct cardiotonic steroids (low doses oleandrin for the brain and ouabain or the heart) was reported for the brain and heart exposed to ischemia-reperfusion (Pasdois et al., 2007; Van Kanegan et al., 2014). Binding of ouabain in low doses to the ATPase protects from toxicity of other cardiotonic steroids (Nesher et al., 2010). Whereas micormolar doses of cardiotonic steroids trigger Ca^2+^ overload and promote apoptosis (Winnicka et al., 2007), nanomolar doses of some of these compounds stimulate proliferation (Winnicka et al., 2010). This anti-apoptotic action may be reflect the changes in affinity of the Na,K-ATPase-ouabain complex to other proteins interacting with the Na,K-ATPase such as PKC, BAX, and Bcl-2 (Lauf et al., 2015). Release of them from the enzyme into the cytosol protects the cells from apoptosis.

On the other hand endogenous ouabain and marinobufagenin act as powerful vasoconstrictors actively participating in development of hypertension (Bagrov and Fedorova, 1998), and have a potential to cause Ca^2+^ overload secondary to the inhibition of the Na^+^ extrusion from hypoxic tissue (Schwinger et al., 1999). At the moment, we clearly know too little about the mechanisms of cytoprotection by cardiotonic steroids to use them effectively as therapeutic agents avoiding deadly side-effects (Washam et al., 2015).

## The role of acute regulation of the NA,K-ATPase in adaptation or irreparable damage at low O_2_ levels

As shown above, the capacity of the Na,K-ATPase to sense and respond to the changes in oxygen availability is immensely diverse with multiple signaling cascades implicated in fine-tuning of the enzyme's activity apart and before terminal ATP depletion is reached. The choice of signaling mechanism as well as the resulting vector and amplitude of the change in the Na,K-ATPase activity depends on the cell type, severity and duration of hypoxia, and is rather species-specific. Strictly speaking, we cannot refer to Na,K-ATPase as the “oxygen-sensitive” protein as all the processes involved in its responding to hypoxia are mediated by secondary products of O_2_ transformation to either gaseous messengers or products of O_2_ reduction.

Several types of response to hypoxia include (i) inhibition by thiol modifications (ii) modulation of the enzyme function/availability at the membrane due to the changes in phosphorylation, and (iii) interaction with nanomolar doses of endogenous cardiotonic steroids.

**Figure 4:**
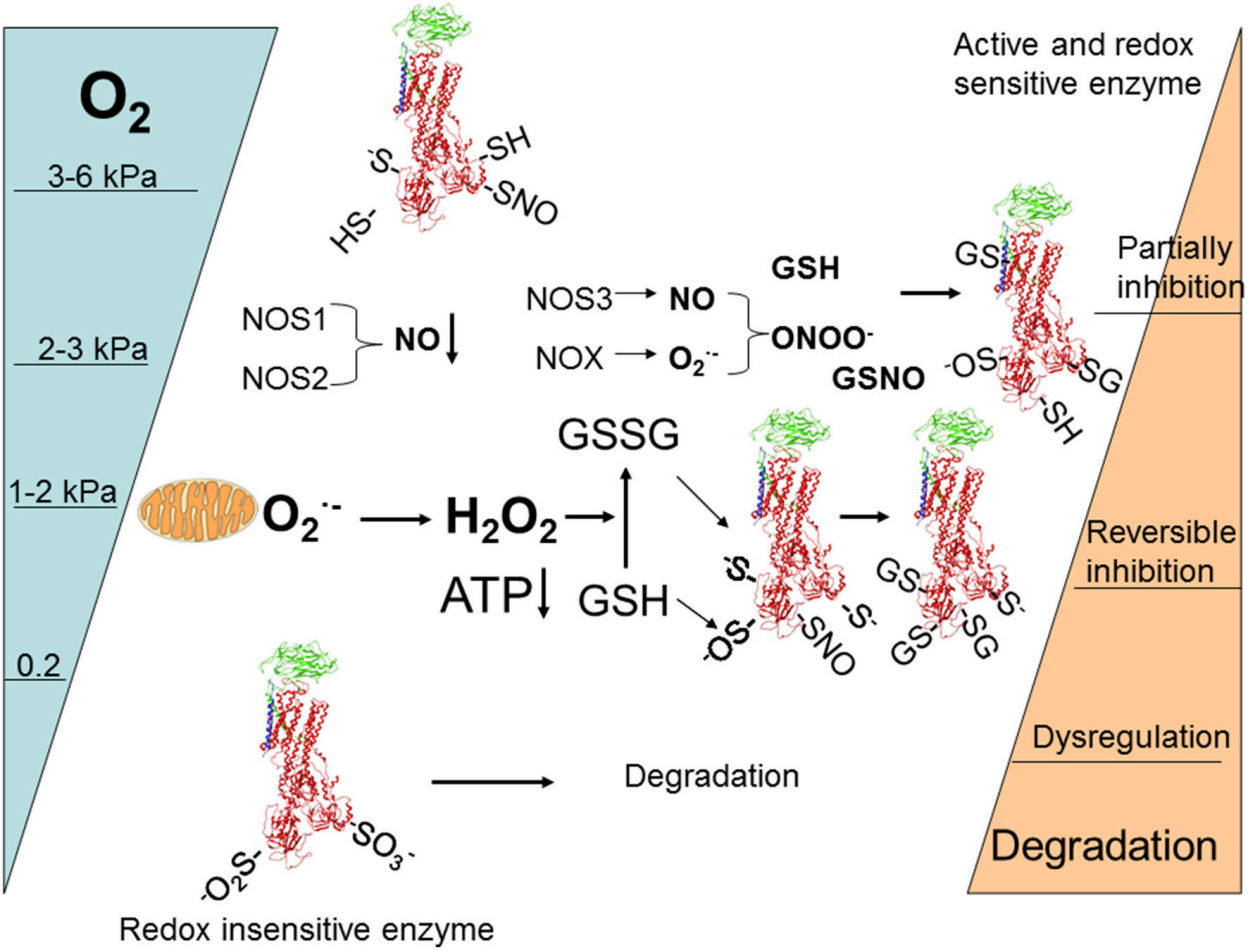
**Schematic representation of O_2_-induced regulation of activity and abundance of Na,K-ATPase in majority of cells and tissues (for details see the Section The role of acute regulation of the Na,K-ATPase in adaptation or irreparable damage at low O_2_ of levels of the review)**.

Diversity of regulatory pathways allows for fine dose-dependent regulation of the enzyme within minutes after the alteration in O_2_ availability. Based on the current knowledge the following scheme of responses may be suggested (Figure 4). Following gradual deoxygenation NO availability in cells expressing NOS1 and NOS2 becomes limited whereas ${\text{O}}_{2}^{•-}$ production by NADPH oxidases is maintained while pO_2_ is above 2–3 kPa (K_d_ for NOXes ~ 2 kPa) (Pacher et al., 2007). This is not the case for tissues in which NOS3 isoform of NO synthase with high affinity for O_2_ prevails (such as vascular endothelial cells). These conditions most likely favor S-glutathionylation of β subunit that is associated with activation of superoxide production by NOXes (White et al., 2009) and α subunits of the Na,K-ATPase due to the gradual accumulation of GSSG (Petrushanko et al., 2012). Further decrease in oxygenation to 1–2 kPa supports uncoupling of electron transfer and triggers mitochondrial ${\text{O}}_{2}^{•-}$ production and accumulation of H_2_O_2_ in the cytosol. Oxidation of GSSG becomes more pronounced and complete inactivation of the enzyme may be achieved as soon as ATP levels decrease below 50 μM (Petrushanko et al., 2012). Further decrease in O_2_ to 0.2 kPa reduces S-glutathionylation and renders regulatory thiols within the α subunit oxidized to sulfinic and sulfonic acid making the enzyme insensitive to the changes in GSSG and ONOO^−^ (Petrushanko et al., 2015). Phosphorylation may support or reduce the probability of complete reversible inactivation by S-glutathionylation of the regulatory cysteines by shifting the equilibrium between the E1 and E2 conformation of the enzyme (see Section Oxygen-Sensitive Phosphorylation).

Survival under hypoxic conditions implies that Na^+^ uptake particularly high in excitable tissues is balanced with equally efficient extrusion of Na^+^ by the pump (Hylland et al., 1997; Nilsson, 2001). Inhibition of the Na,K-ATPase is deadly when not synchronized with the closing of the cation channels and cessation of activity of the animal (channel arrest) (Buck and Hochachka, 1993; Hochachka et al., 1996; Hylland et al., 1997; Silver et al., 1997; Nilsson, 2001; Ross et al., 2006; Wilkie et al., 2008; Dave et al., 2009). If the Na,K-ATPase is suppressed and the channels are not “arrested,” survival is limited to the time of Na^+^/K^+^ gradients dissipation in the brain and in the heart (Hylland et al., 1996, 1997; Silver et al., 1997; Nilsson, 2001). It is tempting to assume that the alterations in free radical production and reversible thiol modifications are involved in the simultaneous regulation of multiple ion transporting systems coupling their activity to the mitochondrial function and ATP availability. However, data providing direct support for this hypothesis are currently missing.

The pilot studies suggest that preservation of redox state in hypoxic heart is sufficient to maintain the Na,K-ATPase activity in Spalax mole rate and trout supporting activity of both species under conditions of critical O_2_ shortage for at least 20 min (Yakushev et al., 2012). S-nitrosylation of the regulatory cysteine residues in the α subunit may also prevent S-glutathionylation and the enzyme inactivation by GSSG in myocardial membranes (Petrushanko et al., 2012). Whether this is the case for some or all anoxia-tolerant species (e.g., Hylland et al., 1997; Nilsson, 2001; Ross et al., 2006; Dave et al., 2009), remains to be clarified.

Maintenance of the transmembrane Na^+^ gradients does not only support neuronal function and heart contractility. It also regulates intracellular Ca^2+^ by controlling the activity of Na/Ca exchanger and that of voltage-gated Ca^2+^ channels. Ca^2+^ transport pathways are by themselves targets regulated by reversible thiol modifications in hypoxic cells (Lehotsky et al., 2002; Wang and Zheng, 2010).

Recent data revealed the existence of intimate link between the activity of the Na,K-ATPase and the gene expression in hypoxic cells and tissues. Two factors that were recently suggested to impact the gene expression under hypoxic conditions include the shift in transmembrane Na/K gradients (Koltsova et al., 2014) and the alteration in HIF1α levels upon binding of the nanomolar concentrations of cardiotonic steroids to their binding site within the Na,K-ATPase (Zhang et al., 2008; Cao et al., 2014). Decrease in HIF1α in hypoxic cells (1% O_2_) in the presence of < 50 nM ouabain is not caused by its degradation but rather by a drop in protein synthesis (Zhang et al., 2008), most likely at the level of translation (Cao et al., 2014). Alterations of HIF1α availability by cardiac glycosides contributes to the modulation of long-term hypoxic responses of the organism. For example digoxin treatment prevents remodeling of pulmonary vasculature underlying development of hypoxic pulmonary hypertension (Abud et al., 2012). It is known that signaling cascades initiated by binding of low doses of cardiotonic steroids to the Na,K-ATPase include modulation of intracellular Ca^2+^ levels and free radical production as well as activation of several kinases. Molecular mechanisms linking these processes to the regulation of HIF1α translation remain unclear.

## Author contributions

AB is the author the general schematics of review, she contributed to all the sections and assembling of the review. AM, PH were contributing to the sections Introduction. Oxygen, and Oxygen Sensing from Evolutionary and Modern Perspectives, Versatility of oxygen sensing. Multiple Signals-Multiple Targets—Multiple Responses—Multiple Outcomes, and Oxygen and Redox-Sensitivity of the Na,K-ATPase. IP contribution were the sections Oxygen and Redox-Sensitivity of the Na,K-ATPase, Coordinated Regulation of Redox-Sensitive Protein Networks by Reversible Thiol, Oxygen-Sensitive Phosphorylation, and Regulation of the Na,K-ATPase Activity by Cardiotonic Steroids. All co-authors discussed the topics and writing the review. All of them agree with the final text.

### Conflict of interest statement

The authors declare that the research was conducted in the absence of any commercial or financial relationships that could be construed as a potential conflict of interest.
